# Relationship between Psychosocial Factors and Physical Activity among Undergraduate Students from a South African University

**DOI:** 10.3390/ijerph21040441

**Published:** 2024-04-03

**Authors:** Chanté Johannes, Nicolette V. Roman, Sunday O. Onagbiye, Simone Titus, Lloyd L. Leach

**Affiliations:** 1 Department of Sports, Recreation, and Exercise Science, University of the Western Cape, Cape Town 7535, South Africa; sonagbiye@frederick.edu (S.O.O.); titusdawsons@sun.ac.za (S.T.); lleach@uwc.ac.za (L.L.L.); 2 Centre for Interdisciplinary Studies of Children, Families and Society, University of the Western Cape, Cape Town 7535, South Africa; nroman@uwc.ac.za; 3 Department of Health and Exercise Sciences, Frederick Community College, Frederick, MD 21701, USA; 4 Centre for Health Professions Education, Faculty of Medicine and Health Sciences, Stellenbosch University, Cape Town 7505, South Africa

**Keywords:** physical activity, psychosocial factors, undergraduate, university, students, mental health, motivation, social support, South Africa, cross-sectional

## Abstract

Psychosocial factors such as mental health, motivation, and social support are key determinants of behavior that play a significant role in physical activity participation. Limited studies have investigated the relationship between psychosocial factors and physical activity among university students in Africa. The aim of this study was to determine the relationship between psychosocial factors and physical activity participation among undergraduate university students at a historically disadvantaged university (HDU) in South Africa. This was a cross-sectional study that used convenience sampling (*n* = 534, majority female, 53.6% with a mean age of 20.69). The study was conducted through an online, self-administered, and hard-copy, valid questionnaire in September 2022. Data on sociodemographic information were collected. Psychosocial factors were assessed using the Depression, Anxiety, and Stress-21 Scale for mental health, the Physical Activity and Leisure Motivation Scale for motivation, and the Perceived Social Support Scale for social support. Physical activity (calculated as MET-min/week) was assessed using the international physical activity questionnaire in short form. Results revealed that almost a third (29%) of undergraduate students were physically inactive, 31.1% were minimally active, and 39.9% were in the health-enhancing category. Physical activity was positively related to stress (r = 0.11, *p* < 0.05) and anxiety (r = 0.10, *p* < 0.05). Motivational factors were positively related to psychological condition and others’ expectations (r = 0.10, *p* < 0.05), and depression and others’ expectations (r = 0.11, *p* < 0.05). Results from this study highlighted that psychosocial factors were related to physical activity participation among undergraduate university students. Psychosocial factors should be considered a coping mechanism when implementing health-promoting strategies.

## 1. Introduction

Physical inactivity is a global public health concern [1], particularly among undergraduate university students [2]. The World Health Organization (WHO) recommends that young adults between the ages of 18 and 25 years should achieve at least 150 min of moderate-intensity physical activity (PA) and/or 75 min of vigorous-intensity PA per week [1]. Globally, PA has been found to be beneficial for young adults, including university students, and may reduce levels of cardiovascular disease, diabetes, weight gain, and sedentary behavior [1,3,4]. Previous studies indicate that insufficient PA among university students is a challenge and that, regardless of the associated health benefits, participation in PA continues to decrease at the tertiary level [5,6,7]. Underlying psychosocial factors such as mental health, motivation, and social support are important determinants of behavior that play a pivotal role in influencing participation in physical activities [8,9,10].

Various psychosocial factors such as social support (family, friends, social integration, and emotional support), psychological well-being (self-efficacy, self-esteem, stress, depression, anxiety, and trust), psychological risk factors (cynicism, vital exhaustion, hopelessness, and depressiveness) and motivation (expectations, readiness, task-orientation, and autonomy) may impact PA behavior [11,12,13]. Previous research indicates that PA is related to mental health [14], social support [15], and motivational factors [16].

According to a previous study discussing the results from the WHO [17], depression affects approximately 300 million people globally and has become the leading cause of disability worldwide. The prevention and treatment of mental health disorders have been described by WHO [1] as a fundamental component of human health. Specifically, depression, anxiety, and stress were identified as global public health concerns, particularly in developing countries [18] and among young adults, such as undergraduate university students [19,20,21,22].

A study of 3092 undergraduate students found that the prevalence and severity of depression increased over the period 2016–2019 [23]. Globally, it was estimated that between 12% and 50% of university students exhibited at least one diagnostic criterion for one or more mental disorders [24], and anxiety was diagnosed in between 12% and 43% of college and university students [25,26,27]. These results were similar to the findings of Bantjes et al. (2019) [28], who suggested that more attention needs to be paid to supporting the psychological well-being of young adults as they transition into tertiary education. Specifically, there was a concern about the mental health of first-year students [23]. First-year students were different from the other undergraduate levels due to the fact that, during their transition into the university environment, they needed support systems to help them adjust academically and mentally to cope with this new tertiary phase of their studies [29]. For this reason, there is a growing body of literature focused on investigating mental health among first-year students [20,30,31]. Furthermore, mental health and motivation are closely intertwined with PA participation, with the former either influencing or reinforcing the latter.

One of the most important psychosocial factors that stimulate and maintain an individual’s engagement in PA is motivation [10,32]. However, the lack of motivation to be physically active has become a critical research topic because of the sedentary lifestyles exhibited by university students [33,34,35,36]. Motivation as a psychosocial factor may facilitate beneficial beliefs about PA and lead to sustained active behavior in university students [9,37]. However, a lack of motivation and willpower negatively impacts participation in leisure-time PA [38]. A clear understanding of motivation may assist in understanding the underlying reasons for individuals being physically active and maintaining this practice or the reason some individuals are less active and sometimes completely withdrawn from PA [36,39]. In addition, social support, such as family and friends, has also been shown to play an important role in PA behavior and motivation [40].

Worldwide, social support, which is seen as a social determinant of PA behavior, was positively related to participation in leisure-time PA among adults [9,41,42,43], especially among university students [9,44]. Social support can be defined as the perception that one is cared for by a supportive social network, such as friends, family, and significant others, which has beneficial effects on mental and physical health [45,46]. Social support in the university environment plays a positive role in maintaining a student’s overall health and well-being [40,44]. Family and friends may offer various types of encouragement, which include emotional, moral, and psychosocial support [47]. However, a considerable number of university students continue to remain physically inactive, and thus, an understanding of how social support from family and friends impacts students’ physical health is important, particularly when developing tailored interventions that promote positive PA behaviors [48]. Social support networks play a crucial role in psychosocial behavior, especially in terms of PA participation [15,49]. Social support is positively related to PA and motivation [15]. Therefore, social support from family and friends is a psychosocial factor that requires further investigation [50].

Physical activity positively influences mental health, motivation, and social support [10,40,44,51,52]. This has been well documented in various studies [52,53,54,55]. Regular PA among university students was shown to alleviate symptoms of depression, anxiety, and stress [56,57,58], improve sleep, mood, motivation, and cardiovascular fitness, reduce body weight and tiredness, help boost energy, and enhance social support [59,60,61]. Therefore, literature on the role of PA as therapy for mental health disorders has surfaced more substantially in recent times [14,62]. However, university students’ PA levels remain insufficient, and thus, the onset of depression, anxiety, stress, lack of motivation, and lack of social support may continue to rise and hinder their PA involvement [20,21,51].

University students in South Africa, in terms of their mental health, motivation, and social support, exhibit a range of characteristics shaped by sociocultural diversity and educational environments. The rich cultural diversity of students from Africa is influenced by social norms, expectations, and preferences regarding PA, mental health, motivation, and social support [63]. Prior research focusing on sub-Saharan African undergraduate students revealed elevated levels of depression, stress, and anxiety, with 48.2% of students identified as experiencing depression [63]. Another study conducted at an African university highlighted a higher prevalence of mental health concerns among students than global studies. The study attributed these mental health concerns to factors such as study program, year of study, workload, sleep quality, gender, and motivation [64]. Previous research at an African university established that social support has a positive influence on mental health [65] and PA motivation of undergraduate university students [46,47]. This suggests that the presence of social support from friends and family acts as a beneficial social network and reduces feelings of depression and anxiety [65]. Furthermore, motivation emerged as a critical factor influencing behavior, as indicated in a South African study where university students displayed robust motivation for self-improvement guided by goal-setting and incentives, which included gaining social support from their family [66]. While these South African studies have delved into various psychosocial aspects among students, such as mental health [63,64,65] and social support [65], these studies frequently centered on one psychosocial factor rather than examining a blend of such factors that could potentially influence health behaviors. Moreover, PA was not explored in these investigations.

Although mental health, motivation, and social support have been previously examined as standalone psychosocial factors [14,46,64,65], there is a scarcity of research within the African context, particularly among undergraduate university students. Relatively little research has been conducted on how psychosocial factors impact a student’s well-being [63]. Furthermore, few studies exist that have investigated PA behaviors among university students in Africa [64,65]. To address the current gap in the literature, this study sought to determine the relationship between psychosocial factors (mental health, motivation, and social support) and PA participation among undergraduate university students.

## 2. Materials and Methods

### 2.1. Study Design

This research adopted a quantitative cross-sectional study design through the application of an online and hard-copy self-administered questionnaire to determine the relationship between psychosocial factors influencing PA participation among undergraduate university students at a historically disadvantaged university (HDU) in the Western Cape province of South Africa. This study focused on a single HDU, which facilitated a comprehensive examination of contextual factors that were directly relevant to the research objective. Universities exist within broader psychosocial contexts, and a targeted investigation enabled a more profound exploration of the distinctive psychosocial dynamics at the HDU. This approach contributed to a deeper understanding of the research context.

### 2.2. Participants and Sampling

Convenience sampling was used to recruit undergraduate university students for this study. University students were readily available within the campus environment, making them convenient participants for the research. The study included undergraduate students from seven faculties, namely Arts and Humanities, Community and Health Sciences, Dentistry, Economic and Management Sciences, Education, Law, and Natural Sciences, at a tertiary institution in the Western Cape province of South Africa. The undergraduate student cohort comprised an approximate population of 19,000 individuals. The power of 0.95 (95%) was calculated to obtain the sample size using Raosoft, Inc. (Raosoft USA, v.7, 2004) software. A total of 375 undergraduate students was calculated as an appropriate sample size. Participant inclusion criteria included being 18 years and older, being a full-time registered student, and providing written consent. Participants were excluded based on being younger than 18 years old, being registered for part-time and semester courses only, or having no written consent provided.

### 2.3. Data Collection Questionnaire

The questionnaire was divided into four sections. Firstly, sociodemographic information was requested (including sex, age, current university faculty, current year of study, relationship status, residence, and disability). Thereafter, PA levels and psychosocial factors, such as mental health, motivation, and social support, were recorded.

### 2.4. Physical Activity: International Physical Activity Questionnaire Shorsat Form

Physical activity was measured using the International Physical Activity Questionnaire Short Form (IPAQ-SF). This form is a self-report questionnaire which assesses PA across seven days. It was developed by the International Consensus Group in 1998. The IPAQ-SF consists of seven items that focus on weekly time spent in vigorous-intensity activity, moderate-intensity activity, and walking. These categories were calculated by multiplying the PA frequency with the duration within each activity category. The total weekly PA was determined by adding the three categories of activity listed above. In addition, the questionnaires also recorded the amount of sitting time [67]. The level of PA was quantified using the metabolic equivalent of task (MET)—reported as MET-min/week [68]. Within this study, the variable MET–min/week expresses weekly metabolic engagement in vigorous, moderate, and walking physical activities [68]. The questionnaire consists of three levels (or categories) that determine the level of PA: Category 1: inactive (low activity level), Category 2: minimally active (moderate activity level), and Category 3: health-enhancing physical activity (HEPA) (high activity level). The IPAQ-SF was proven to be reliable in this study, with test–retest scores for vigorous-intensity PA (0.85), moderate-intensity PA (0.86), walking (0.77), and sitting (0.76).

### 2.5. Mental Health: Depression, Anxiety, and Stress Scale–21

The Depression, Anxiety, and Stress Scale 21 (DASS-21) is a quantitative measure of distress. It is an abbreviated version of the longer DASS-42, which contains 42 items [18]. It assesses three separate but interrelated subscales—depression, anxiety, and stress—as experienced during the last week on a 4-point Likert scale (0–3) where “0” signifies “did not apply” and “3” indicates “very much or most of the time”. The DASS-21 is not meant for clinical diagnosis [69]. It is important to note that the results derived from this scale were used to measure the prevalence of depression, anxiety, and stress of undergraduate students and not for diagnostic purposes. The scores of the DASS-21 were doubled to correspond to scores on the 42-item DASS in order to interpret the severity of each emotional state [70]. The DASS-21 subscales were found to be reliable in this study. For online: depression (0.916), anxiety (0.846), and stress (0.863). For hard copy: depression (0.904), anxiety (0.855), and stress (0.856). For the entire sample: depression (0.909), anxiety (0.856), and stress (0.870).

### 2.6. Motivation: Physical Activity and Leisure Motivation Scale

The Physical Activity and Leisure Motivation Scale (PALMS) is based on both empirical and theoretical approaches to understanding PA and leisure motivation. This scale was developed by Rogers and colleagues in 2008 [71]. The questionnaire consists of 40 items with eight subscales, namely (1) mastery, (2) physical condition, (3) affiliation, (4) psychological condition, (5) appearance, (6) others’ expectations, (7) enjoyment, and (8) competition/ego. The original tool consisted of a 5-point Likert scale (1–5) consisting of 40 items: 1 = strongly disagree, 2 = disagree, 3 = neutral, 4 = agree, and 5 = strongly agree. For this study, the Likert scale was adapted to a 4-point scale (1–4) consisting of 1 = strongly disagree, 2= disagree, 3 = agree, and 4 = strongly agree. This scale was adapted to reduce vagueness when answering a particular statement by removing the “neutral” option. The PALMS subscales were found to be reliable in this study. For online: mastery (0.804), physical condition (0.893), affiliation (0.873), psychological condition (0.883), appearance (0.859), other’s expectations (0.767), enjoyment (0.878), and competition and ego (0.833). For hard copy: mastery (0.837), physical condition (0.878), affiliation (0.788), psychological condition (0.860), appearance (0.872), other’s expectations (0.744), enjoyment (0.823), and competition/ego (0.825). For the entire sample: mastery (0.825), physical condition (0.886), affiliation (0.823), psychological condition (0.869), appearance (0.868), other’s expectations (0.754), enjoyment (0.845), and competition/ego (0.832).

### 2.7. Social Support: Perceived Social Support from Family and Friends Scale

The Perceived Social Support from Family (PSS-Fa) and Friends (PSS-Fr) scale was developed by Procidano and Heller in 1983 [72]. This tool is a quantitative measure of the social support from family and friends. Previous research used this tool to investigate the factors of social support among university students and the influence of these factors on PA engagement [73,74]. The original tool consisted of a 3-point Likert scale (1–3) of 20 items for friends and 20 items for family: 1 = Don’t know, 2 = No, 3 = Yes. This tool was adapted for this study to a 4-point Likert scale (1–4) consisting of 1 = strongly disagree, 2 = disagree, 3 = agree, and 4 = strongly agree. The PSS scale was found to be reliable in this study. For online: family (0.894) and friends (0.846). For hard copy: family (0.880) and friends (0.9.11). For the entire sample: family (0.862) and friends (0.888).

### 2.8. Procedure

The online questionnaire was built using the Google Forms platform. Requisite permissions were secured from the researchers through email to employ the questionnaire for this study. Thereafter, approval was received from the corresponding university. The online link was then distributed through the university communication email channel, where student emails were filtered to only include full-time undergraduate students. Between September and November 2022, prospective participants responded to an email sent to their university-affiliated email addresses explaining the study and inviting them to voluntarily participate. The link to the questionnaire was accessible via computer, smartphone, and tablet at the convenience of the student. The first page of the questionnaire contained the informed consent form, which provided information on the study, such as the procedures, potential risks, benefits, and researcher contact details. Participant anonymity and confidentiality were ensured as no student-identifying information was recorded. Participants provided their informed consent by clicking the “continue to the questionnaire on page 2, Section 2” button and proceeding to the next screen, where the questionnaire began. Should a student not wish to partake in the study, after reading the information and consent form, the questionnaire would conclude with a “thank you for your time” message. Thereafter, the student would no longer have access to the remaining sections of the questionnaire. A total of 189 students completed the online questionnaire.

**Figure 1:**
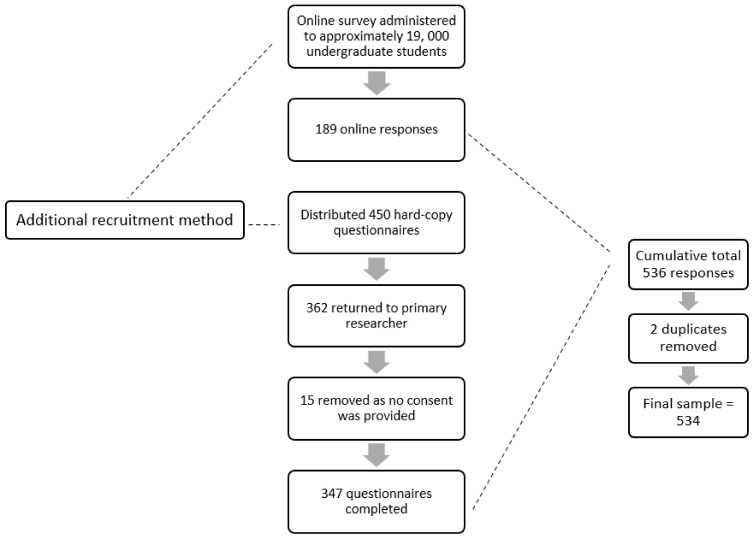
Data collection procedure.

In order to increase the participant response rate, hard-copy questionnaires were distributed as well. Regarding the distribution of the hard-copy questionnaires, the primary researcher briefed two research assistants regarding the objectives of the study and the inclusion and exclusion criteria for the recruitment process. Students were required to create a unique identification code in order for the researchers to remove duplicates between the hard-copy and online questionnaires. The online and hard-copy questionnaires contained the exact same information and questions. A total of 450 hard-copy questionnaires were distributed throughout the student cafeteria to eligible participants. Choosing a high-traffic area, such as the student cafeteria, was a convenient location to recruit students. Three hundred and sixty-two (362) hard-copy questionnaires were returned. Fifteen (15) questionnaires were removed as participants did not provide consent. A total of 347 completed hard-copy questionnaires were obtained. A cumulative total amount of 536 responses was received from participants through the online and hard-copy recruitment process. Two duplicates were removed, therefore obtaining a final sample of 534. Therefore, this study reports on two merged sample sets. The data collection procedure is visually depicted in Figure 1 below.

### 2.9. Statistical Analysis

The Statistical Package for the Social Sciences (SPSS) version 28.0 (Chicago, IL, USA) was applied for data analysis. Data were collected, coded, and cleaned for errors using the double-entry method within Microsoft Excel (version 16, 2019). The sample characteristics were analyzed using frequencies and percentages, as well as means and standard deviations for quantitative data.

Tests of normality for the PALMS subscales using Shapiro–Wilks indicated a violation of normality for all subscales (*p* < 0.05). Tests of normality for the DASS-21 subscales indicated a violation of normality for all three subscales, as well as the DASS-21 (Shapiro–Wilks = *p* <0.05). Tests of normality for the PSS indicate that the subscale Family had statistical normality (Shapiro–Wilks = *p* > 0.05), while the Friends subscale did not show statistical normality (Shapiro–Wilks = *p* < 0.05). However, previous research has indicated that the samples do not have to be normally distributed given a sufficient sample size [75,76,77].

In order to test differences in PA between two groups based on sociodemographic information, an independent samples *t*-test was used. The variables included sex, relationship status, disability, and place of residence. One-way ANOVA was used to test differences between multiple groups when there were more than two categories. In this case, the variables were faculty and year of study. For faculty, none of the groups (i.e., per faculty) had normality of data (Shapiro–Wilks = *p* < 0.00). Levene’s test for homogeneity of variance was significant (*p* < 0.00); hence, there was no homogeneity of variance. For year of study, none of the groups (i.e., study year) had normality of data (Shapiro–Wilks = *p* < 0.00). Levene’s test for homogeneity of variance was significant (*p* < 0.00); thus, there was no homogeneity of variance. Therefore, ANOVA analyses can withstand minor violations of the assumption of homogeneity of variances [76].

As assessed by Levene’s test for equality of variances, the assumption of homogeneity of variances was violated for total MET-min/week for the sex variable (*p* = 0.02). As such, the Welch *t*-test statistic was interpreted.

Pearson’s correlation was used to examine the relationship between psychosocial factors, such as mental health, motivation, social support, and PA levels, where significance levels were accepted at *p* < 0.05. The analysis broadened the understanding of the interplay between these variables and their relation to PA levels.

Logic regression analysis was used, where the IPAQ was the outcome variable, categorized into inactive, minimally active, and HEPA. The predictor variables were psychosocial factors, using the subscales from PALMS, DASS-21, and PSS. The results of the regression analysis shed light on the predictive power of these psychosocial factors in determining PA levels. This comprehensive interpretation provided insights into the observed associations and their significance in addressing the research objectives.

### 2.10. Ethical Considerations

All participants agreed to participate in this study by providing written consent. Ethics approval for this study was granted by the Humanities and Social Sciences Research Ethics Committee at the University of the Western Cape, reference number HS21/10/24, prior to the start of this investigation.

## 3. Results

### 3.1. Information of Participants

Table 1 presents the sociodemographic results of the participants. Five hundred and thirty-four (534) undergraduate students (53.6% female) participated in this study. The participants’ ages ranged from 18 to 42 years, with a mean age of 21.11 (SD = 2.71) years.

The majority of the students were enrolled in the Faculty of Community and Health Sciences (26.8%), were in their first year of study (38.6%), were not in a relationship (55.8%), did not have a disability (98.1%), and lived off campus (89.9%). Of those in a relationship, 43.22% (n = 102) stated that their intimate partner supported their being physically active. Only 0.2% (1.0) indicated that their disability hindered their ability to be physically active.

### 3.2. Physical Activity Levels

Table 2 shows the varying PA levels of undergraduate university students. In terms of vigorous-intensity PA, students engaged on average 1.87 days per week, with an average daily duration of 45.89 min, totaling 1275.13 MET minutes per week. Moderate-intensity PA occurred on average 1.68 days per week, with a duration of 35.01 min and a weekly volume of 446.20 MET minutes. Walking activity was observed to occur approximately 4.95 days per week, with an average daily duration of 56.05 min and a total volume of 1077.32 MET minutes per week. Sedentary behavior was notable, with students spending an average of 247.72 min per day sitting.

Table 3 presents the PA results according to sociodemographic information. Males (40.7%) and females (39.2%) were approximately equal in terms of engaging in HEPA. PA across the university faculties indicated that the majority of students who were physically inactive were in Dentistry (35.4%). More than a third of the students in the faculty of Arts and the faculty of Community and Health Sciences were minimally active: 36.1% and 35.0%, respectively. The majority of students in the faculties of Education (39.8%), Economic and Management Sciences (47.8%), Law (42.3%), and Natural Sciences (40.3%) participated in HEPA. In terms of the PA levels across the year of study, most students participated in HEPA, with the exception of the fourth-year students (35.9%), who were mainly physically inactive. Students who were either in a relationship (36.0%) or single (43.0%), as well as those living on campus (40.7%) and off-campus (39.8%), indicated that they participated in HEPA.

Females had slightly higher total volume of PA per week than males. However, this was not a statistically significant difference (t (530.77) = −0.602, *p* = 0.55).

The assumption of homogeneity of variance was met for disability (*p* = 0.07), relationship status (*p* = 0.46), and place of residence (*p* = 0.78). In terms of disability, there was no statistical difference in PA between disabled and able-bodied participants (t (532) = 0.70, *p* = 0.48). There was no significant difference in PA between participants in an intimate relationship and those who were not in a relationship (t (532) = −0.975, *p* = 0.33). Lastly, in terms of place of residence, there was also no significant difference between participants who lived on campus and those off campus (t (532) = 0.497, *p* = 0.62).

One-way ANOVA was run to establish whether there were differences in the total volume of PA per faculty and year level, as measured by MET-min/week. The results showed no significant differences between faculty groups (F (6, 527) = 2.06, *p* = 0.057). The results also showed no significant differences between the different years of study (F (4, 529) = 2.0, *p* = 0.090).

### 3.3. Relationship between Psychosocial Factors and Physical Activity

Table 4 shows the results of the correlation between psychosocial factors and the total volume of PA. Significant correlations were found between the mental health subscales and total volume of PA, specifically anxiety (r = 0.10, *p* < 0.05) and stress (r = 0.11, *p* < 0.05). Furthermore, significant correlations were found between psychological condition and others’ expectations (r = 0.10, *p* < 0.05) and between depression and others’ expectations (r = 0.11, *p* < 0.05). No significant correlations were found between the motivation subscales and total volume of PA, specifically mastery (r = −0.00, *p* > 0.05) and physical condition (r = 0.00, *p* > 0.05). No significant correlation was found between the social support subscales, family, and total volume of PA (r = 0.00, *p* > 0.05) and friends (r = −0.1, *p* > 0.05).

Table 5 depicts the odds ratios for the three categories of PA. The odds ratio for all variables across the three categories appears to be similar in value for all of the variables in each model. The test for parallel lines confirms the assumption of equal odds (χ^2^ (13) = 18.84, *p* = 0.128). The final model significantly predicts the dependent variable beyond the intercept-only model (χ^2^ (13) = 26.60, *p* < 0.05). However, the deviance goodness-of-fit test suggests the model may not fit well due to multiple expected cells with zero frequencies (χ^2^ (1043) = 1125.90, *p* = 0.037). Therefore, caution is needed when interpreting this statistic. The model’s Nagelkerke’s pseudo-R-square value is 0.055, which indicates a 5.5% improvement in predicting PA based on the predictors in this model.

Table 6 shows the contribution of each psychosocial variable when predicting the PA category. The first assumption, when testing with ordinal logistic regression, was multicollinearity. Tolerance values were greater than 0.1, indicating that multicollinearity was not a concern. The second assumption was that of proportional odds. This assumption was tested by running separate binary logistic regression analyses. Dummy variables were created for the three categories of inactive, minimally active, and HEPA to test this assumption. Odds ratios were used to measure the relationship between the predictor variable (psychosocial factors—mental health, motivation, and social support) and outcome variables (PA). The test for parallel lines indicated that the assumption of equal odds had been met (χ^2^ (13) = 18.84, *p* = 0.128).

Psychological condition, enjoyment, and competition/ego were statistically significant. Therefore, there is a relationship between these psychosocial subscales and PA. An increase in psychological condition motivation was positively associated with an increase in the odds of being in a higher PA category, with an odds ratio of 1.16, 95% CI [0.913, 1.104], (Wald χ^2^ (1) = 12.24, *p* < 0.01). An increase in competition/ego motivation was positively associated with an increase in the odds of being in a higher PA category, with an odds ratio of 1.09, 95% CI [1.022, 1.151], (Wald χ^2^ (1) = 6.46, *p* < 0.05). Enjoyment showed a negative contribution, with a decrease in the motivation enjoyment decreasing the odds of being in a higher PA category with an odds ratio of 0.90, 95% CI [0.810, 0.985], (Wald χ^2^ (1) = 6.06, *p* < 0.05). None of the other psychosocial factors were significant in the prediction model.

## 4. Discussion

This study aimed to determine the relationship between psychosocial factors and PA participation among undergraduate university students at a historically disadvantaged university (HDU) in South Africa. Psychosocial factors such as mental health, motivation, and social support were determinants of PA behavior among students who are at risk of leading sedentary lifestyles. Particularly and recently in South African universities, psychosocial wellness has become characterized as a pressing public health issue due to mental health challenges among students [78], lack of motivation [36], and lack of social support networks [47]. Data from this study highlight the relationship between the psychosocial factors of mental health, motivation, social support, and PA. In this study, a considerable number of undergraduate university students were identified as physically inactive. In addition, the research revealed significant relationships between stress, anxiety, and PA engagement. Specifically, a positive correlation was observed between psychological condition and others’ expectations. The study also revealed a positive relationship between depression and others’ expectations. However, no significant relationship was found between support from family and friends and the level of PA participation. These results highlight the interplay among psychosocial factors that contribute to PA behaviors of undergraduate university students.

### 4.1. Physical Activity Levels

In today’s fast-paced and typically sedentary society, the importance of PA in maintaining overall well-being cannot be emphasized enough [79]. The results from this study indicated that approximately a third (29%) of undergraduate university students were physically inactive. These results are consistent with previous research, which found that about one-third of undergraduate students were inactive, particularly in their first year at university [68,80]. It has further been reported that participation in PA among young adults has been low in sub-Saharan African populations [81]. Numerous factors may contribute to the high prevalence of physical inactivity among undergraduate university students. The demanding nature and pressure of academic studies may lead to students prioritizing their studies over PA [82]. The first year of university represents a transition period where students are adjusting to their new routines, which may have an impact on their PA habits [50]. Additional factors may include a lack of time and motivation, a lack of accessible places, and a lack of financial resources [83].

A study focusing on South African university students revealed that 33% participated in low levels of PA [82]. Our study reported similar results, showing that students in the Arts and Dentistry faculties were inactive, at 31.9% and 34.5%, respectively. More specifically, physically inactive students were in their third (31.4%) and fourth (23.9%) year levels of study. Previous authors found that, among South African individuals, only 27.8% were vigorously active [83]. However, our results indicated that 31.1% of students were minimally active, and 39.9% engaged in vigorous PA. Therefore, according to our results, although students were engaging in health-enhancing physical activities, a large proportion of students were physically inactive and were not meeting the WHO PA recommendations [1]. This is concerning because the health behaviors of students at the university level often have a long-lasting effect on their adult years, which may also negatively impact their mental health [79]. Various factors may contribute to the current results, which indicate that a significant number of students are inactive. Differences in PA preferences may lead to some students engaging in vigorous-intensity PA, while others may prefer a sedentary lifestyle [84]. A lack of PA awareness and education [85] could suggest that students may not be fully aware of the recommended PA levels from the WHO [1]. In addition, students facing mental health challenges might lack motivation to engage in PA. This could be influenced by factors such as social isolation, fear of judgment, and a lack of energy [86].

### 4.2. Mental Health and Physical Activity

Previous research has shown that mental health can influence PA levels and, with an impaired mental state, PA performance may decrease [87]. However, this study revealed that stress and anxiety were significantly related to PA engagement. It should be emphasized that a causal relationship cannot be made; nevertheless, it is plausible that students who engaged in PA more frequently experienced elevated levels of stress and anxiety. This contradicts previous literature, which indicated that a negative relationship between mental health and PA exists [87]. A prior study found that students experienced depressive disorder (24.7%), and 20.8% reported an anxiety disorder, which negatively influenced student wellness [28]. Moreover, and similar to the results in this study, research on mental health and PA found that PA levels were positively correlated with an increase in well-being (*p* < 0.0001) [87]. Therefore, PA may be viewed as a protective factor against mental disorders [87]. Research has suggested that PA should be explored as a coping mechanism to reduce stress and anxiety, which may positively influence mood [88]. Physical activity (PA) has been reported as having antidepressant effects [84]. Therefore, with increased PA participation, undergraduate students may encounter lower symptoms of depression, anxiety, and stress [56,57,58]. Engaging in regular PA is particularly important for performing academically at a university level, as there are many stressors that students encounter [79]. These stressors could include academic pressure, a lack of belonging, feeling overwhelmed and fatigued by studying, and a lack of social relations [85].

The academic journey of undergraduate university students is usually characterized as a stressful experience coupled with high levels of anxiety [79]. Previous research indicates that the mental health of university students in sub-Saharan Africa is a priority, given its strong association with academic performance [24,86]. By participating regularly in PA, students have an outlet to manage symptoms of stress and anxiety. Consequently, this enables students to be better equipped to focus on their academic performance [14,89,90]. In the South African context, where job security is tenuous, good academic performance may serve as an asset for employability [91,92,93]. 

### 4.3. Motivation and Physical Activity

The relationship between motivation and PA has been well-researched [10,94]. According to previous research, motivation has often been associated with participating in high levels of PA [39].

Psychological condition, as a psychosocial motivator, centers on an individual’s capacity to relax, manage stress, reduce internal and external pressures, and redirect their focus away from various concerns [32,95]. Others’ expectations, also considered a psychosocial motivator, revolve around factors such as people encouraging an individual to engage in PA [96]. This study found that there was a positive correlation between psychological condition and others’ expectations: the higher the psychological condition as a motivational factor, the higher the expectations of others become. This result was similar to a study that investigated PA motives among a cohort of South African students across three universities [97]. Their results indicated a positive relationship between PA and the motives of revitalization, enjoyment, challenge, social recognition, and competition. The findings of this study concurred with their results and suggested that the expectations of others, such as exercise groups or teammates, may create a sense of accountability. Students may use peers as accountability partners to maintain a PA routine [98]. Once this routine develops, it becomes an expectation to participate in the fitness group in order to maintain their social connections.

This study further indicated that depression and others’ expectations had a positive relationship. The higher the expectations, the higher the symptoms of depression. Some societal norms encourage regular PA participation to maintain mental and physical health [51]. Although PA may be beneficial in alleviating symptoms of depression [99], the pressure to meet the expectations of others may be overwhelming [100]. The perceived obligation to engage in regular PA may create stress and feelings of guilt, especially if individuals do not meet the expectations of others [101,102]. Thus, a student with depression may be physically active, but only to the extent of gaining approval from others with expectations [103].

Hilger-Kolb, Loerbroks, and Diehl (2020) [94] suggest that motivational barriers such as fear of failure to master a new skill or task, fear of judgment from peers, a lack of affiliation and belonging, low self-esteem and ego, discontent with appearance, dissatisfaction with psychological and physiological conditions, and fear of competitiveness to succeed, negatively affect PA participation among university students [16,94,104].

### 4.4. Social Support and Physical Activity

Social support is another critical factor that influences an individual’s participation in PA [9]. Social support can be classified as a social determinant of PA among university students [105]. Participating in PA with others can create a sense of camaraderie and accountability, making it more enjoyable and sustainable [106]. In addition, family and peer behaviors act as contributing factors that may influence the extent of social support for PA engagement [107]. Conversely, a lack of motivation to be physically active could be attributed to a lack of social support [9,108]. This study found no relationship between support from family and friends and PA participation, however.

The university setting is characterized as a diverse environment [109]. As such, not all students would be interested in PA, as some may deem it important for overall well-being while others may consider it non-essential [110]. This is the reason various students may not share the same interests and preferences when participating in PA. Another characteristic of students attending university is the display of autonomy [111]. A new level of independence can provide students with the opportunity to formulate decisions based on their goals and preferences instead of succumbing to peer pressure and expectations [111]. However, previous research has suggested that the support of family and friends is important in enhancing a student’s psychosocial well-being, especially within South African universities [86,91]. Understanding the different types of social support of family and friends is essential to developing tailored and culturally appropriate intervention programs to promote PA participation among students [48].

### 4.5. Strengths and Limitations

This is the first known paper to report on the relationship between psychosocial factors influencing PA participation among undergraduate university students in Africa. This research highlights the limited exploration of psychosocial factors influencing PA behavior among undergraduate university students, especially within an African context. A limitation exists regarding the convenience sampling method that was utilized. It is difficult to generalize the results, and the cross-sectional nature of the study means that it is impossible to determine causal relationships. Future research should consider using random sampling methods and developing longitudinal studies that would enable causal relationships to be determined.

While this study made efforts to minimize bias in this study, it is important to acknowledge that the inclusion of both online and hard-copy formats of the questionnaire may introduce potential biases into our analysis, such as differences in user behavior between online and hard-copy formats. Despite best efforts to mitigate these biases through rigorous data validation and analysis procedures, it is encouraged that readers interpret the findings thoughtfully.

While the IPAQ-SF serves as a valuable tool for assessing PA participation, a limitation arises from its self-administered nature, which has the potential to lead to varied perceptions of PA. Thus, the reliance on subjective responses may introduce bias. To address this limitation and enhance accuracy, additional instruments such as accelerometry, which objectively measures PA, would provide a more comprehensive and reliable evaluation of PA levels among students. While the DASS-21 is a suitable tool to screen depression, anxiety, and stress, it is important to recognize a limitation in its application. This tool is designed to identify students who are at risk of being affected by these conditions but is not intended for diagnostic purposes. To establish a formal diagnosis, additional tools specifically designed for diagnostic purposes should be used. The DASS-21, PALMS, and PSS scales were self-administered questionnaires, and thus, students’ responses may have been subjective.

### 4.6. Recommendations

Based on the results obtained from this study, the following recommendations are provided to improve undergraduate student PA engagement while considering the psychosocial factors that may influence PA behavior. Firstly, mental health workshops (for stress and anxiety management) could be implemented to promote the connection between mental health and PA, in addition to promoting PA as a coping mechanism. Physical activities for these workshops could include yoga, Pilates, and meditation classes. Secondly, motivation could be encouraged through motivational campaigns, where students develop their personal goals related to PA. Physical activities to enhance motivation could include team sports (such as soccer) and/or group classes (such as aerobics or dance). Fitness challenges and competitions as PA enhancement strategies could be used by students to support and motivate one another to achieve their fitness goals. Lastly, social support in the form of peer support workshops could be offered, in which group classes involving endurance activities could be used to encourage social interaction and PA participation. Endurance activities include walking, jogging, hiking, and/or aerobics. These activities could create positive social support networks that may improve the PA levels of undergraduate students. The results obtained from this study provide a basis for future researchers to implement strategies to improve mental health, motivation, and social support factors that are imperative for the overall well-being of students.

In order to gain a more profound understanding of PA participation, qualitative studies could be conducted to gain a deeper understanding of the relationship between psychosocial factors and PA. Furthermore, an exploration and investigation of sociodemographic factors in relation to participation in PA could be considered. To encourage PA participation among undergraduate students, a holistic approach is needed. This may involve collaborative efforts with support services and a focus on diversifying PA initiatives, introducing peer-led programs, and integrating technology to track PA levels.

Researchers aiming to advance the exploration of the relationship between psychosocial factors and PA may consider a multifaceted approach. To enhance the accuracy and depth of PA assessment, researchers could investigate the incorporation of objective measures, such as accelerometry, alongside widely used self-administered tools such as the IPAQ-SF. Comparative studies employing both subjective and objective measures could provide a more robust understanding of PA levels. In addition, using a mixed-method approach could provide a comprehensive understanding of PA participation that could allow for data triangulation and a more profound exploration of students’ behaviors. Lastly, longitudinal studies investigating the relation between psychosocial factors and changes in PA levels over an extended period could contribute valuable insights that may inform the development of targeted interventions.

## 5. Conclusions

The results from this study highlight the relationship between psychosocial factors and participation in PA among undergraduate university students. Psychosocial factors impacting mental health, such as stress and anxiety, were significantly related to PA. Motivational factors, such as psychological conditions and others’ expectations, were significantly related to depression. Physical activity (PA) was associated with mental health, including depression, anxiety, and stress. Therefore, future research on mental health should investigate PA interventions as a coping strategy among undergraduate university students. By understanding the relationship between psychosocial factors and PA, researchers could develop tailored interventions that enhance undergraduate students’ holistic well-being.

## Figures and Tables

**Table 1:** Sociodemographic information of participants.

Variables	Category	N	%
Sex	Male	248	46.4
	Female	286	53.6
Faculty	Community and Health Sciences	143	26.8
	Education	83	15.5
	Arts	72	13.5
	Economic and Management Sciences	69	12.9
	Natural Sciences	67	12.5
	Law	52	9.7
	Dentistry	48	9.0
Year of study	1	206	38.6
	2	150	28.1
	3	105	19.7
	4	64	12.0
	5	9	1.7
Relationship status	In a relationship	236	44.2
	Single	298	55.8
Disability status	Has a disability	10	1.9
	Does not have a disability	524	98.1
Residence	Lives on campus	54	10.1
	Lives off campus	495	89.9

**Table 2:** Physical activity levels of undergraduate university students.

Category		Mean	SD
Vigorous-intensity physical activity	Frequency of vigorous-intensity physical activity (days per week)	1.87	1.96
	Duration of vigorous-intensity physical activity (minutes per day)	45.89	57.11
	Volume of vigorous-intensity physical activity (MET minutes per week)	1275.13	1948.54
Moderate-intensity physical activity	Frequency of moderate-intensity physical activity (days per week)	1.68	1.91
	Duration of moderate-intensity physical activity (minutes per day)	35.01	50.16
	Volume of moderate-intensity physical activity (MET minutes per week)	446.20	840.29
Walking activity	Frequency of walking activity (days per week)	4.95	2.59
	Duration of walking activity (minutes per day)	56.05	68.44
	Volume of walking activity (MET minutes per week)	1077.32	1440.65
Sedentary behavior	Sitting time (minutes per day)	247.72	170.29

**Table 3:** Differences in PA categories between groups.

		Physically Inactive		Minimally Active		HEPA*	
Variable	Category	N	%	N	%	N	%
Sample	n = 534	155	29.0	166	31.1	213	39.9
Sex	Male	70	28.2	77	31.0	101	40.7
	Female	85	29.7	89	31.1	112	39.2
Faculty	Arts	23	31.9	26	36.1	23	31.9
	Community and Health Sciences	34	23.9	50	35.0	59	41.3
	Dentistry	17	35.4	15	31.3	16	33.3
	Education	26	31.3	24	28.9	33	39.8
	Economic and Management Sciences	23	33.3	13	18.8	33	47.8
	Law	12	23.1	18	34.6	22	42.3
	Natural Sciences	20	29.9	20	29.9	27	40.3
Year of study	1	57	27.7	69	33.5	80	38.8
	2	41	27.3	42	28.0	67	44.7
	3	33	31.4	33	31.4	39	37.1
	4	23	35.9	19	29.7	22	34.4
	5	1	11.1	3	33.3	5	55.6
Relationship status	In a relationship	73	30.9	78	33.1	85	36.0
	Single	82	27.5	88	29.5	128	43.0
Disability status	Has a disability	4	40.0	3	30.0	3	30.0
	Does not have a disability	151	28.8	163	31.1	210	40.1
Residence	Lives on campus	14	25.9	18	33.3	22	40.7
	Lives off campus	141	29.4	148	30.8	191	39.8

HEPA* indicates health-enhancing physical activity.

**Table 4:** Correlation between psychosocial factors and total volume of PA.

	Variables	1	2	3	4	5	6	7	8	9	10	11	12	13
1.	Total MET-min/week	-												
2.	Mastery	−0.00	−											
3.	Physical condition	0.00	0.80 **	−										
4.	Affiliation	0.01	0.52 **	0.38 **	−									
5.	Psychological condition	0.04	0.68 **	0.79 **	0.41 **	−								
6.	Appearance	−0.01	0.72 **	0.80 **	0.43 **	0.70 **	−							
7.	Others’ expectations	−0.01	0.21 **	0.05	0.43 **	0.10 *	0.16 **	−						
8.	Enjoyment	−0.02	0.78 **	0.76 **	0.50 **	0.77 **	0.70 **	0.14 **	−					
9.	Competition/ego	0.04	0.41 **	0.19 **	0.53 **	0.19 **	0.30 **	0.63 **	0.36 **	−				
10.	Depression	0.08	−0.24 **	−0.31 **	−0.10 *	−0.28 **	−0.23 **	0.11 *	−0.28 **	−0.03	−			
11.	Anxiety	0.10 *	−0.25 **	−0.35 **	−0.09 *	−0.31 **	−0.25 **	0.13 **	−0.28 **	0.01	0.83 **	−		
12.	Stress	0.11 *	−0.24 **	−0.31 **	−0.10 *	−0.27 **	−0.22 **	0.08	−0.26 **	−0.06	0.85 **	0.86 **	−	
13.	Family	0.00	0.31 **	0.32 **	0.16 **	0.33 **	0.29 **	−0.00	0.34 **	0.03	−0.39 **	−0.31 **	−0.33 **	-
14.	Friends	−0.01	0.28 **	0.29 **	0.24 **	0.29 **	0.27 **	−0.11 *	0.29 **	−0.00	−0.31 **	−0.28 **	−0.25 **	0.40 **

* Correlation is significant at the 0.05 level; ** Correlation is significant at the 0.001 level.

**Table 5:** The odds ratio for psychosocial factors and PA levels.

	B (Parameter Estimates)			Exp (B) (Odds Ratio, OR)		
Predictor	Physically Inactive	Minimally Active	HEPA*	Physically Inactive	Minimally Active	HEPA
Mastery	0.102	−0.103	0.010	1.107	0.902	1.010
Physical condition	−0.059	0.082	−0.032	0.943	1.086	0.969
Affiliation	−0.035	0.094	−0.054	0.966	1.098	0.947
Psychological condition	−0.173	0.017	0.136	0.841	1.018	1.145
Appearance	0.002	0.017	−0.015	1.002	1.017	0.985
Others’ expectations	0.033	0.028	−0.054	1.033	1.028	0.948
Enjoyment	0.163	−0.068	−0.081	1.177	0.934	0.922
Competition/ego	−0.081	−0.031	0.103	0.922	0.970	1.108
Depression	0.020	0.011	−0.028	1.020	1.011	0.972
Anxiety	−0.033	0.024	0.004	0.968	1.024	1.004
Stress	−0.003	−0.059	0.059	0.997	0.942	1.061
Family	−0.001	−0.002	0.004	0.999	0.998	1.004
Friends	0.018	−0.003	−0.014	1.018	0.997	0.986

HEPA* indicates health-enhancing physical activity.

**Table 6:** Logistic regression revealing the association between psychosocial factors and the prediction of PA categories.

					95% CI for Exp(B)	
	Estimate (B)	Exp (B)	Std. Error	Sig.	Upper Bound	Lower Bound
Mastery	−0.041	0.960	0.043	0.334	1.044	0.867
Physical Condition	0.009	1.009	0.048	0.854	1.104	0.913
Affiliation	−0.016	0.984	0.030	0.600	1.043	0.926
Psychological Condition	0.145	1.156	0.041	<0.001	1.236	1.076
Appearance	−0.003	0.997	0.037	0.928	1.070	0.925
Others’ Expectations	−0.037	0.963	0.030	0.220	1.023	0.904
Enjoyment	−0.107	0.898	0.044	0.014	0.985	0.810
Competition/Ego	0.084	1.087	0.033	0.011	1.151	1.022
Anxiety	0.018	0.976	0.031	0.547	1.037	0.915
Stress	0.034	1.019	0.032	0.275	1.083	0.954
Depression	−0.024	1.035	0.027	0.374	1.088	0.983
Family	0.003	1.003	0.009	0.777	1.021	0.985
Friends	−0.017	0.984	0.009	0.081	1.002	0.966

## Data Availability

The data presented in this study are available on request from the corresponding author. The data are not publicly available due to ethical restrictions.
